# Symmetrical drug-related intertriginous and flexural exanthema (SDRIFE): two atypical case reports

**DOI:** 10.1186/1939-4551-8-S1-A84

**Published:** 2015-04-08

**Authors:** Eduardo Longen, Ana Carolina D'onofrio-Silva, Marcelo Vivolo Aun, Marisa Rosimeire Ribeiro, Laila Sabino Garro, Jorge Kalil, Pedro Giavina -Bianchi, Antonio Abílio Motta, Violeta Regnier Galvão

**Affiliations:** 1 University of São Paulo, Brazil

## Background

The symmetrical drug-related intertriginous and flexural exanthema (SDRIFE) is a delayed-type hypersensitivity drug reaction (HDR) that causes symmetrical erythematous lesions in flexural areas, including buttocks and groin, which arise following exposure to drugs, especially beta-lactams. The involvement of palms and soles is rare and, until now, it has only been described after exposure of amoxicillin. We hereby report a patient with SDRIFE and involvement of the palms and soles after taking cephalexin and another patient who developed SDRIFE after exposure to doxycyclin.

## Methods

Literature review and case description.

## Results

Two patients referred to our outpatient clinic specialized in HDR for evaluation of adverse drug reactions compatible to SDRIFE. The first patient was a man, 50 years of age, with a history of erythematous lesions with blisters in flexural areas, including groin, associated with lesions of the palms and soles 20 hours after he had started taking cephalexin to an orbital cellulitis. We performed intradermal skin tests with penicillin, cefazolin and ceftriaxone, which resulted negative. We also performed oral drug provocation test (DPT) with amoxicillin and cefuroxime, in order to provide therapeutical options with different betalactams. The patient was also advised to avoid taking cephalexin and other betalactams with similar group side chains. The second patient was a woman, 64 years of age, with a history of skin lesions in flexural areas, including groin, compatible to SDRIFE. She had started taking doxycyclin to treat erysipelas four days before the first lesions appeared. She was hospitalized for five days and got better with prednisone 0.5mg/kg and fexofenadine. The antibiotic was switched to clindamycin and she was told to avoid tetracyclines for the future.

## Conclusions

SDRIFE is a rare HDR and betalactams are the main cause of these reactions. However, as far as we know, these were the first patients presenting palm and sole involvement due to cephalexin and a typical SDRIFE after taking doxycyclin.

## Consent

Written informed consent was obtained from the patient for publication of this abstract and any accompanying images. A copy of the written consent is available for review by the Editor of this journal.

